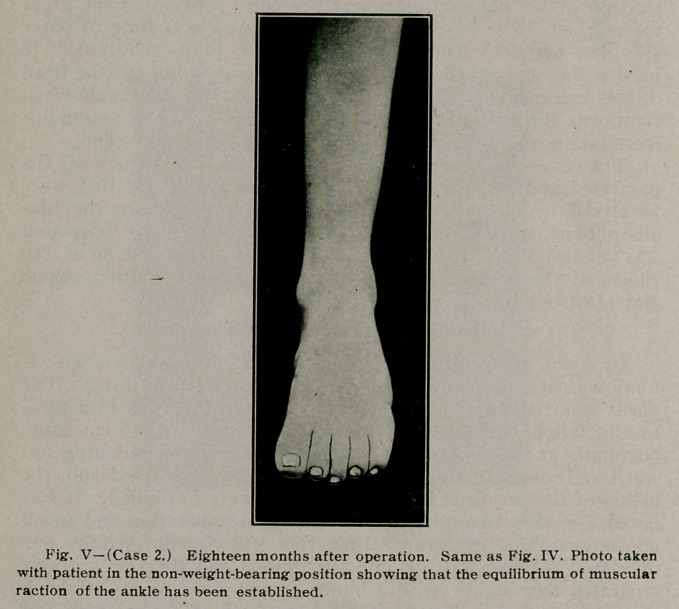# A Consideration of Two Distinctly Different Types of Talipes Equino Varus with Illustrative Cases

**Published:** 1909-11

**Authors:** Roland O. Meisenbach

**Affiliations:** Buffalo, N. Y.; Clinical Instructor in Orthopedic Surgery, University of Buffalo


### A Consideration of Two Distinctly Different Types of Talipes Equino Varus with Illustrative Cases.

By ROLAND O. MEISENBACH, M. D., Buffalo, N. Y.
Clinical Instructor in Orthopedic Surgery, University of Bnffalo.

THERE are two distinct types of talipes equino-varus or club-foot which are commonly met with—namely, the congenital and paralytic. The *congenital* is usually caused by the malposition of the fetus in utero, whether by direct pressure of the foot on the uterine wall or by fibrous bands and adhesions. There are other theories but space will not permit, nor is it the object to enter into the etiology at this time. This malposition allows the different bones of the foot chiefly the astragalus, os calcis and cuboid, to grow in a faulty position, so that when muscular traction enters into the case the muscles act at such angles that there is no chance for the foot to gain its normal position. In some cases the muscular traction is so great in the latter months of pregnancy that the bones have already assumed a fixed shape. In the other cases this traction is not as severe, and it is possible to correct by manual force and manipulation with retentive apparatus after the child is one or two months old. The diagnosis in the congenital type is not difficult. The treatment in the congenital type, depending upon the conditions, is manipulative, apparatus, tenotomy, osteotomy, or a combination of the above, depending upon the amount of distortion of the bones and the amount of muscular traction.

Fig. I—(Case 1). Congenital club-foot with bony hypertrophy before operation; a) Head of astragalus; (b) Bursa; (c) Anterior portion of os calcis.

The *paralytic* club-foot is usually of gradual onset, resulting from infantile paralysis or paresis of some of the muscles about the ankle joint. After the paralysis has run its course in some cases all the muscles regain their normal power except a few. The peroneus longus and peroneus brevis most frequently remain paralyzed; in some cases the anterior tibial group or the posterior tibial group may remain paralyzed or be in a state of paresis. An accurate diagnosis with the view to the method of procedure in the paralytic form of club-foot is not as easy as in the congenital, for the reason that it is essential to know the true state of all the muscles if one wishes a functionating foot without a relapse as the result. In many cases, some of the muscles are seemingly paralyzed due to over-stretching, and if the case is not carefully studied the over-stretched tendons may be mistaken for paralyzed. In some cases a number of different procedures may be elected in the same case, but the ultimate result depends upon the judgment of the orthopedic surgeon. The prevention of the deformity depends upon its early recognition and upon the early application of corrective apparatus. When the case is advanced and the deformity marked, the operative procedure is necessary.

Fig. II—(Case 1). Congenital club-foot fourteen months after operation. Same as Fig. I.

TENDON TRANSPLANTATION.

Before doing tendon transplantation for club-foot, the operator should have studied the case carefully and in no instance should the operation be performed without this. The operator should have a clear knowledge of the condition of all the tendons involved; that is, he should recognise the actually paralyzed tendons and those that are partially paralyzed or in a state of paresis from those that are not functionating due to *over-stretching*. Before the operation, the operator should understand clearly those tendons which he intends to transfer and those which he intends to bisect and transfer; for instance, if the anterior tibial tendon is over-stretched due to tension of the tendo Achillis, it would be wrong to jeopardise the former by surgical interference, because as soon as the traction of the tendo Achillis is removed, the anterior tibial tendon will regain its function normally; also if the tendo Achillis is to be bisected and two independent muscles established, it requires surgical and mechanical judgment to know whether to transplant one-half or two-thirds of the tendo Achillis. Establishing the true condition of overstretched tendons is best done by the method of Jones, who suggested additional pressure upon the tendon, and if any response is the result, the tendon probably is paralysed from overstretching. Electrical tests are of little value. In order to obtain the best results in tendon transference, the post-operative treatment is important; that is, the tension should be taken from the transferred tendon until it is strong enough to assume its new function. This is best done by the wearing of apparatus or the arrangement of the shoe so that the tension will not be placed upon the transplanted tendons. Much of the success of tendon transference is dependent upon the post-operative treatment by the orthopedist and many of the failures reported are due to lack of its recognition.

To illustrate the two different types of cases, I am herewith reporting in detail the method of procedure in each instance. Naturally, each case of the different types must be studied individually so that the cases reported illustrate the method of procedure in (1) the congenital type; (2) the paralytic type of club-foot.

*Case I. Congenital Club-Foot, Five Years Standing, with Distortion of the Bones, Articular Surfaces, Tendons and Muscles.*

In this case it was necessary to do a bone operation but before this could be done, a tenotomy was necessary. The case presented is a lad, five years old, very nervous and unruly, emaciated, teeth decayed, and unable to walk slowly on account of the unstable condition of the right foot, which was permanently fixed in the extreme position of talipes equino varus. The posterior part of the os calcis was tilted upward and back, while the anterior portion extended downward. The astragalus had rotated outward and the neck had grown downward. The cuboid had rotated inward and downward. The tubercle of the scaphoid was palpable in the normal position of the internal malleolus. The internal malleolus was buried deep in soft tissue and could not be palpated. The tendo Achillis was tense and the plantar fascia firm and contracted. There was some motion in the toes but none in the tibio-astragaloid or other joints of the foot. On the upper surface of the foot, which in this case would be the anterior portion of the os calcis and the upper portion of the cuboid, a large bursa, 4 by 5 cm. and 3 cm. thick. had formed. This acted as a natural pad while the child walked (See Fig. 1.)

BONE OFERATION.

On June 3, 1908, under ether and aseptic precautions, the foot was thoroughly and forcibly manipulated with the view of correcting as much of the varus as possible before making the incisions. The varus was slightly corrected but not to an appreciable amount, owing to the hypertrophy of some of the bones. The foot was then thoroughly scrubbed again and a fasciatomy of the plantar fascia done, and the tendo Achillis divided subcutaneously. A semi-lunar incision was then made over the

Fig. III—Club-foot shoe for post-operative treatment.

bursa and the latter resected. It was found that the bursa was adherent by fibrous bands in all directions to the periosteum of the os calcis, cuboid and astragalus. The cuboid was found to be hypertrophied and the head of the os calcis also considerably enlarged. With an osteotome, a cuneiform section of bone was removed from the os calcis a few cm. in back of the calcaneo-cuboidal articulation. I chose this as the point of election for the osteotomy so that I would not ankylôse the calcaneo-cuboidal articulation. The common operation is to do the osteotomy in the cuboid instead of the os calcis, but it was not done in this case for the reason stated above. The foot was then forcibly brought into position and a plastic skin operation done, then the case put up in plaster. Staphylococcus infection developed with multiple abscesses in the shoulder, right knee and in the region of the former bursa. This retarded convalescence somewhat but was readily controlled. The abscess in the clavicle showed a pure staphylococcus culture and therefore an autogeneous vaccine was prepared and the case readily controlled by serum therapy.

POST-OPERATIVE TREATMENT OF THE FOOT.

Keeping in mind that relapsed club-feet are not uncommon occurrences, after the plaster was removed the patient was put into a club-foot shoe (Fig. III). This shoe was corrected at

Fig. IV—(Case 2) Paralytic club-foot with the peroneus longus and peroneus brevis totally paralyzed and the tibialis anticus in a state of paresis due to overstretching. (a) Point at which the external portion of the tendo Achillis was transplanted into the peroneus longus. (b) Peroneus brevis reefed. Photo shows foot before operation.

intervals of months so as to compensate for the muscular development and to prevent a recurrence. After eight months, the patient was allowed to gradually omit the shoe and was given a night shoe to wear, gradually omitting the steel club-foot shoe at intervals during his walking. The patient rapidly became less nervous and more controllable; he grew fat and is able to walk as an ordinary child with the exception of a slight limp, due to the fact that compensation has not as yet been completely established (See Fig. II).

*Case II. Illustrates Club-Foot caused by infantile paralysis.*

The patient is a bright girl, six years old, who had infantile paralysis at the age of two, affecting her entire limb. The child regained the power of the limb and all of the muscles, except some of the muscles in the leg. The mother noticed that gradually the child's foot was growing in the shape of a club-foot, and the child was walking on the external portion of her ankle, at which point a large callus had formed. A brace, purchased at an instrument store, had been worn without results.

Physical examination of the foot showed the foot held in a position of talipes equino varus (Fig. IV). The tendo Achillis was tense and pulling at an obtuse angle and holding the foot in the position of equinus. The anterior tibial tendon at first appeared paralysed, as did also the extensor proprius hallucis. Jones's test was applied and found positive. Considerable contraction of the plantar fascia was present. Flexion of the great toe marked. The posterior tibial tendon was in a state of paresis. The peroneus longus and brevis were totally paralysed. A hard callus was noted over the point corresponding to the head of the calcaneum. The *x*-ray showed that the astragalo-tibial joint was thrown open and beginning changes in the astragalus were taking place. The texture of the bones was atrophic.

The case was carefully studied and the mechanics of the paralysed and non-paralysed tendons as well as those that were paralysed by virtue of overstretching, as for instance the tibialis anticus, were given due consideration before the operation was undertaken. The result of the case depended upon the choice of the tendons to be transplanted as well as the technic and after-treatment.

### OPERATION—TENDON TRANSPLANTATION.

In April, 1906, under ether and aseptic precautions, a fasciatomy was first performed and the varus corrected by force. The varus was temporarily overcome so that the foot was in a position of talipes equinus. A somewhat spiral incision, 16 cm. long, beginning at the insertion of the tendo Achillis and radiating upward and outward was made, the skin was retracted and the bellies of the peroneus longus and brevis were examined. It was noted that these were in a state of fatty degeneration and to all appearances that of paralysed muscles. The tendo Achillis was then split in half, well up into the muscle and the two portions separated thoroughly with a blunt dissector so as to establish two independent muscles. The outer half was sectioned and brought into the position of the peroneus longus. The remaining half was then severed from its attachment and the foot brought into dorsi flexion. I here deviated from the classical operation and reefed the peroneus brevis, bringing it tight so that it would hold the foot in position and thereby act as a stay. The outer portion of the tendo Achillis was then inserted by means of quilt sutures of silk into the peroneus longus, care being taken not to injure the tendon sheaths. The foot was then put up in plaster in a slightly pronated position so as to take off all strain from the transferred tendon. The skin incision was closed with subcutaneous sutures; the incision healed by first intention. The convalescence was uneventful and after three weeks it was noticed that the child could move the toes somewhat better, and that the great toe was not flexed as much as before. After eight weeks the patient was taken out of the plaster and placed in a paralytic club-foot brace, with a shoe built up on the outside so as to cause pronation while walking, in order to take off the strain from the newly formed perinei group. The upright of the brace was also bent so as to hold the pronated foot in place; the child was allowed the use of her foot. After six months it was noticed that the foot remained in position without apparatus and practically all motions of the foot were restored, and that equalisation of muscular traction had been cor-

Fig. V—(Case 2.) Eighteen months after operation. Same as Fig. IV. Photo taken with patient in the non-weight-bearing position showing that the equilibrium of muscular raction of the ankle has been established.

rectly established. Owing to the articular surface of the astragalo-tibial joint, the patient was made to wear apparatus for more than a year. After eighteen months had elapsed, it was noticed that the anterior portion of the foot was rapidly developing and the foot was growing stronger. Intermittent use of the apparatus was advised, owing to the fact that the child was very lively and a sprain of the ankle was to be avoided.

Result—a perfect functionating and useful foot (See Fig. V).

CONCLUSION.

These two cases cited illustrate very clearly the two types of club-foot most commonly met with in orthopedic practice, and owing to their severity are sometimes looked upon as hopeless or incurable. In the above article it was not my intention to give the detailed treatment of the intermediate stages of these two types, of which many could be cited, but to show that the two types are distinct and require entirely different technic, that the results depend upon careful diagnosis before the operation is undertaken and that the two types require entirely different operative procedure; also, that the post-operative treatment is important and if conscientiously carried out, will result in fewer cases of failures reported in the literature; the family should also be advised that the after-treatment is essential over a long period, otherwise the foot may approach the old position.

140 Allen Street.

## Figures and Tables

**Fig. I f1:**
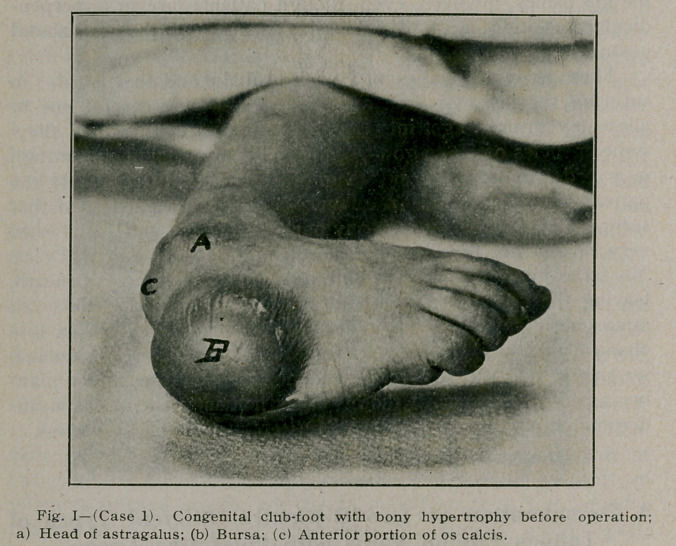


**Fig. II f2:**
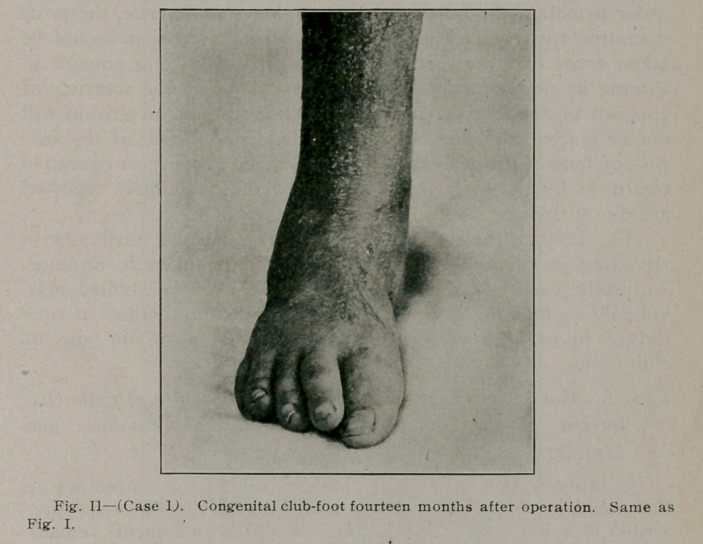


**Fig. III f3:**
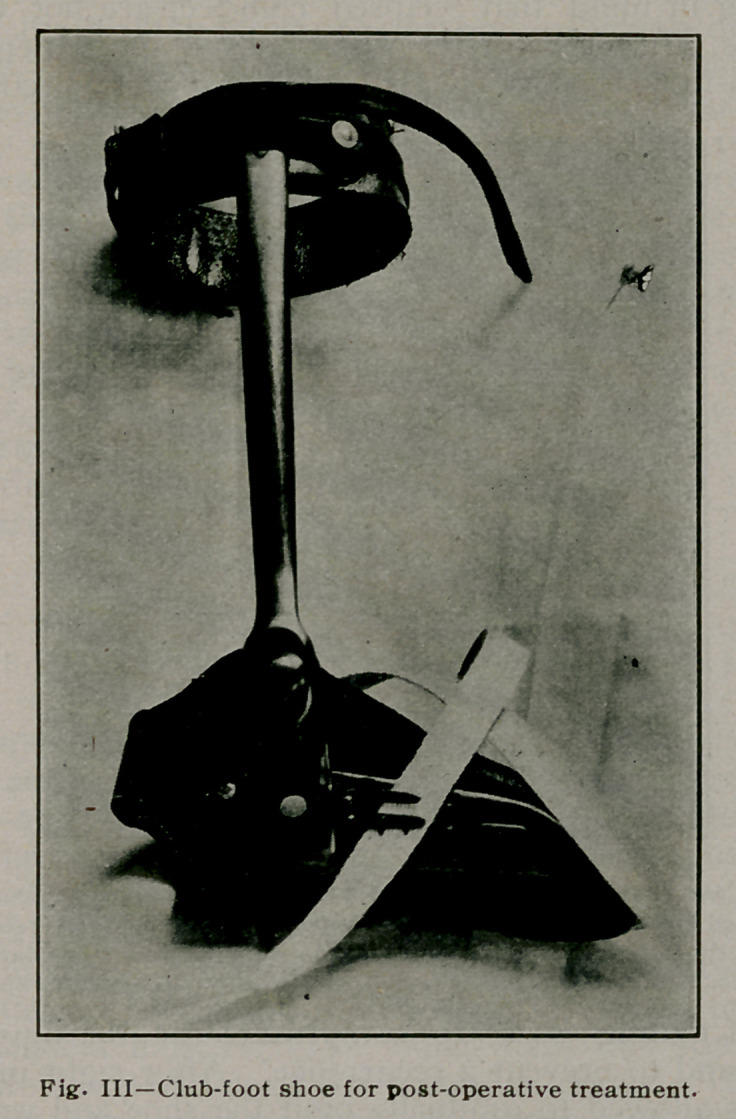


**Fig. IV f4:**
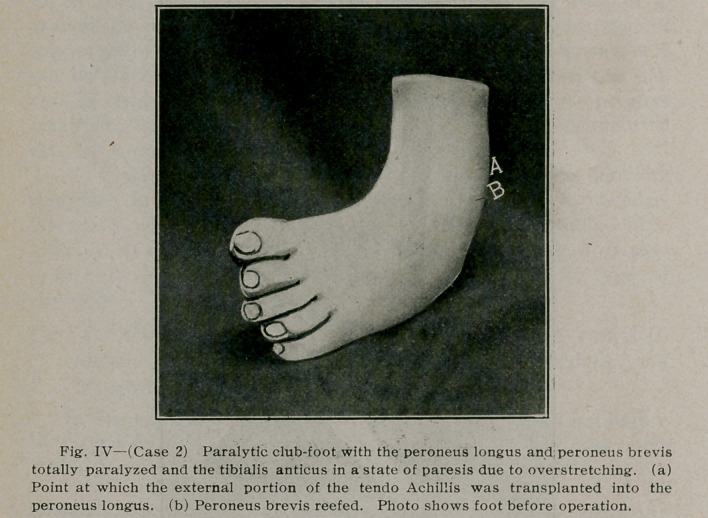


**Fig. V f5:**